# Impact of student choice on academic performance: cross-sectional and longitudinal observations of a student cohort

**DOI:** 10.1186/1472-6920-13-26

**Published:** 2013-02-19

**Authors:** Michael J Murphy, Rohini DeA Seneviratne, Lynda Cochrane, Margery H Davis, Gary J Mires

**Affiliations:** 1 Centre for Undergraduate Medicine, Medical Education Institute, Dundee, UK; 2 Centre for Medical Education, Medical Education Institute, Dundee, UK; 3 Dundee Epidemiology and Biostatistics Unit, Division of Clinical and Population Sciences and Education, University of Dundee, Dundee, UK; 4 Department of Biochemical Medicine, Ninewells Hospital & Medical School, DD1 9SY, Dundee, UK

## Abstract

**Background:**

Student choice plays a prominent role in the undergraduate curriculum in many contemporary medical schools. A key unanswered question relates to its impact on academic performance.

**Methods:**

We studied 301 students who were in years 2 and 3 of their medical studies in 2005/06. We investigated the relationship between SSC grade and allocated preference. Separately, we examined the impact of ‘self-proposing’ (students designing and completing their own SSC) on academic performance in other, standard-set, summative assessments throughout the curriculum. The chi-squared test was used to compare academic performance in SSC according to allocated preference. Generalised estimating equations were used to investigate the effect of self-proposing on performance in standard-set examinations.

**Results:**

(1) Performance in staff-designed SSC was not related to allocated preference. (2) Performance in year 1 main examination was one of the key predictors of performance in written and OSCE examinations in years 2, 3 and 4 (p<0.001). (3) The higher the score in the year 1 examination, the more likely a student was to self-propose in subsequent years (OR [CI] 1.07 [1.03-1.11], p<0.001). (4) Academic performance of students who self-proposed at least once in years 2 and/or 3 varied according to gender and year of course.

**Conclusion:**

In this study, no association was observed between allocated preference and SSC grade. The effect of self-proposing on academic performance in standard-set examinations was small. Our findings suggest instead that academically brighter students are more likely to design their own modules. Although student choice may have educational benefits, this report does not provide convincing evidence that it improves academic performance.

## Background

### General

Student choice has long featured in undergraduate learning, but recently has played a more prominent role than previously within the curriculum in British medical schools. This followed recommendations made by the General Medical Council in *Tomorrow’s Doctors*, first published in 1993 [1]. In that document, allowing students to express their individuality and to explore areas of particular interest to them was seen as crucial to harness their engagement with the process of reform. Intercalated degree courses, and electives, whilst laudable, were deemed insufficient on their own to provide the degree of choice envisaged – student-chosen modules would have to be embedded throughout the entire curriculum, forming “a thread running throughout the course rather than confined to a discrete period” [1]. In subsequent editions of *Tomorrow’s Doctors*, the proportion of curriculum time that must be devoted to student-chosen modules has been progressively reduced, but their stated purpose remains “the intellectual development of the student through exploring in depth a subject of their choice” [2].

Student-selected components (SSC), as they are now known, provide students with experience of learning in small groups, and opportunities to develop self-directed learning skills. Some medical schools allow students to design their own modules (within the boundaries of the curriculum), permitting an unrivalled degree of student ownership over learning. However, their accommodation within the undergraduate curriculum has required substantial changes [3]. SSC programmes are administratively complex, and standardisation of assessment is problematic [4]. Moreover, providing students with genuine choice from diverse programmes of high-quality modules presents an ongoing challenge in many institutions [5]. Evidence of added value is therefore important.

A key unanswered question relates to the impact of student choice on academic performance. If it could be shown that student choice resulted in better academic performance, this would be important evidence of benefit. In the current report, we posed the question in cross-sectional and longitudinal ways: (1) Is the performance of students in SSC affected by the allocated preference (i.e. whether the allocated module is their first or other choice)? (2) Does the experience of designing and completing their own modules affect the performance of students in other summative assessments?

### Student-selected components in Dundee

**Figure 1:**
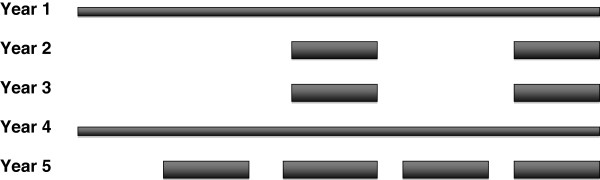
**SSC in Dundee.** Student-selected components in the Dundee undergraduate medical curriculum.

The student-selected ‘thread’ of the Dundee undergraduate curriculum is outlined in Figure 1. Students perform longitudinal exercises in years 1 and 4 of a 5-year curriculum, e.g. literature reviews, data analyses, etc. In years 2, 3 and 5, SSC are undertaken in discrete blocks, which occupy approximately one-third of curriculum time. The undergraduate curriculum was administratively divided into three phases: Phase 1 (year 1), Phase 2 (years 2 and 3) and Phase 3 (years 4 and 5). In Phase 2, sixteen weeks were devoted to SSC, all of which were either two or four weeks long. Students were allowed to choose from a menu of staff-designed SSC, and/or to design (‘self-propose’) their own modules. No restriction or obligation was placed on them to self-propose; many completed both staff-designed and student-designed modules. However, it was made clear to them that self-proposing was the only way of guaranteeing allocation to a preferred topic or unit. Allocation to staff-designed SSC was based on iterative computer-based matching of ranked student preferences with available places.

### Student-designed modules (‘self-proposed’ SSC)

The self-proposal process has been described previously [6]. Briefly, self-proposing students were required initially to contact potential supervisors and, after consultation with them, to submit a written proposal detailing the educational objectives and learning outcomes of the proposed module. The detailed substance of individual self-proposals was arrived at independently and the course-work was not linked to the work of previous students. Proposals were subsequently modified as appropriate after discussion with the SSC Convenor and their supervisor, in a series of iterations of educational objectives in terms of detail and focus. Thus, self-proposing students had, before they started, invested substantial time and effort in establishing realistic, feasible objectives and, more generically, in defining the educational content of their module.

## Methods

### Students

Students who were in years 2 and 3 of the curriculum in 2005/06 were studied. 301 students undertook 1551 SSC modules (including SPSSC modules). 188 (62.5%) were female; 113 (37.5%) male. Students who had returned to year 4 of their medical studies after completing an intercalated degree were excluded from the analysis of performance in year 4 summative examinations (although their data were included for the analysis of performance in earlier years of the medical curriculum). Graduate entrants were also excluded from analysis. The skills gained during completion of undergraduate degrees may have confounded our analyses, because they might affect (a) the academic performance of graduate students in assessments [7] and (b) their threshold for self-proposing.

For the analysis of academic performance in standard-set examinations, students were categorised by whether or not they had, at any time during years 2 and 3 of their medical studies, designed their own SSC module (‘self-proposed’). Thus, students were categorised as either ‘ever self-proposed’ if they had, or ‘never self-proposed’ if they had not.

### Assessment

SSC assessment was summative and separate from other parts of the curriculum. Formal SSC assessment was divided into three sections, with corresponding weightings: 70% of the marks were allocated to written course-work; 15% to interest/motivation; 15% to reliability. Grade descriptors were generic, reflecting the heterogeneous nature of the SSC programme. Standard-set examinations consisted of written or online examinations and, from year 2, objective structured clinical examinations (OSCE). A modified Angoff approach was used to standard-set these examinations. Briefly, this is a test-centred, criterion-referenced approach that relies on expert judgements of how borderline candidates will perform [8].

### Statistical methods

The chi-squared goodness-of-fit test was used to compare academic performance in staff-designed SSC according to allocated preference.

Generalised estimating equations (GEE) were used to investigate the effect of undertaking a self-proposed SSC on performance in standard-set examinations throughout the curriculum. Examination scores in adjacent years were more highly correlated than those more than one year apart, so the AR (1) correlation matrix structure was used. The best-fit model was identified using the Quasi Likelihood under Independence Model Criterion (QIC). The score attained in the year 1 main examination was the first available measure for each student, and was used as a covariate in GEE analyses. It was compared in the relevant self-proposal groups (‘ever’ and ‘never’) in order to investigate base-level differences in performance in standard-set examinations. Only data from first attempts were included in the analyses.

The data reported here were collected as part of routine quality assurance of undergraduate medical training in our institution. Ethical principles were adhered to in the retrieval and analysis of the data, and a waiver obtained from the University of Dundee Research Ethics Committee.

## Results

### Phase 2 SSC grades according to preference

SSC grades awarded to students allocated to staff-designed SSC were compared according to preference (first-, second-, third-, and other choice allocations). Performance was not related to allocated preference, either in females (p=0.79) or males (p=0.49) (Table 1).

**Table 1:** SSC grades by allocated preference and gender

**Gender**	**Grade**		**Preference**				**Total**
			**1**	**2**	**3**	**4 or lower**	
**Female**	A	N	58	24	22	31	135
p=0.786		%	25.9%	19.5%	27.5%	24.2%	24.3%
	B	N	108	60	39	61	268
		%	48.2%	48.8%	48.8%	47.7%	48.3%
	C or less	N	58	39	19	36	152
		%	25.9%	31.7%	23.8%	28.1%	27.4%
	Total	N	224	123	80	128	555
		%	100.0%	100.0%	100.0%	100.0%	100.0%
**Male**	A	N	22	12	12	15	61
p=0.494		%	16.2%	18.2%	28.6%	17.6%	18.5%
	B	N	61	30	19	44	154
		%	44.9%	45.5%	45.2%	51.8%	46.8%
	C or less	N	53	24	11	26	114
		%	39.0%	36.4%	26.2%	30.6%	34.7%
	Total	N	136	66	42	85	329
		%	100.0%	100.0%	100.0%	100.0%	100.0%
**Overall**	A	N	80	36	34	46	196
p=0.566		%	22.2%	19.0%	27.9%	21.6%	22.2%
	B	N	169	90	58	105	422
		%	46.9%	47.6%	47.5%	49.3%	47.7%
	C or less	N	111	63	30	62	266
		%	30.8%	33.3%	24.6%	29.1%	30.1%
	Total	N	360	189	122	213	884
		%	100.0%	100.0%	100.0%	100.0%	100.0%

Student selected components: academic performance according to allocated preference, in females, males and overall.

### Factors affecting performance in standard-set examinations including self-proposal status

Student numbers varied from one year to the next, due, for example, to termination of studies, withdrawal for health reasons, etc. Of the 205 students for whom progression information was available, 101 (49.3%) self-proposed at some stage during Phase 2, i.e. in year 2 and/or in year 3. 65 of these (64.4%) were female. The higher the score in the year 1 main examination, the more likely a student was to self-propose: OR [CI], P 1.07 [1.03-1.11, p<0.001].

The main predictors of performance in written and OSCE examinations in years 2, 3 and 4 were performance in the year 1 main examination, and year of course (p≤0.001 for these associations, Table 2). Gender was also a significant factor in OSCE examinations; females scored more highly than males. Self-proposal status had no impact on examination performance as a main effect, although an interaction with gender was observed in written (p=0.006) and OSCE examinations (p=0.015) (Table 2).

**Table 2:** Standard-set examinations in years 2, 3 and 4: factors affecting academic performance

**Factor**	**Significance**
**Written**	
Self-proposed in Year 2 and/or Year 3 (SP)	0.141
Year of course	<0.001***
Year 1 examination score	<0.001***
Gender	0.612
Year of course × SP	0.305
Year of course × Gender	0.675
Year of course × Year 1 examination score	<0.001***
SP × Gender	0.006**
SP × Year 1 examination score	0.258
Gender × Year 1 examination score	0.399
**OSCE**	
Self-proposed in Year 2 and/or Year 3 (SP)	0.964
Year of course	0.001**
Year 1 examination score	<0.001***
Gender	0.031*
Year of course × SP	0.375
Year of course × Gender	0.383
Year of course × Year 1 examination score	0.003**
SP × Gender	0.015*
SP × Year 1 examination score	0.648
Gender × Year 1 examination score	0.026*

Significance of factors in GEE analysis of examination scores (Years 2, 3 and 4).

OSCE: Objective Structured Clinical Examination.

***significant at p<0.001.

**significant at p<0.01.

*significant at p<0.05.

Table 3 summarises student performance in standard-set examinations from years 1 through 4, by gender and self-proposal status. (In year 1 there was no OSCE examination). The only comparisons significant at the 5% level were in OSCE exams at years 3 and 4: in both cases, males who self-proposed scored more highly than those who did not. There were no similar findings in females.

**Table 3:** Examination scores (as %) in years 1 to 4, by gender and self-proposal status

**Year**	**Gender**	**SP**	**Mean (SE) N**	**p**	**Mean (SE) N**	**p**
			**Written**		**OSCE**	
1	F	No	75.3 (1.01) 34	0.438	NA	
		Yes	76.4 (1.01) 64		NA	
	M	No	77.0 (1.26) 27	0.627	NA	
		Yes	77.9 (1.34) 31		NA	
	Overall	No	76.0 (0.79) 61	0.455	NA	
		Yes	76.9 (0.81) 95		NA	
2	F	No	69.0 (1.21) 34	0.186	73.5 (0.95) 34	0.456
		Yes	70.8 (0.76) 65		74.4 (0.78) 65	
	M	No	69.3 (1.40) 28	0.526	72.3 (0.91) 28	0.550
		Yes	68.1 (1.24) 31		71.4 (1.16) 31	
	Overall	No	69.1 (0.91) 62	0.461	72.9 (0.66) 62	0.585
		Yes	70.0 (0.66) 96		73.4 (0.66) 96	
3	F	No	72.2 (1.17) 34	0.272	76.7 (0.81) 34	0.171
		Yes	73.7 (0.78) 65		78.1 (0.61) 65	
	M	No	72.4 (1.22) 28	0.908	73.6 (0.97) 28	0.093
		Yes	72.2 (1.19) 31		75.8 (0.85) 31	
	Overall	No	72.3 (0.84) 62	0.379	75.3 (0.65) 62	0.013*
		Yes	73.2 (0.66) 96		77.4 (0.51) 96	
4	F	No	78.4 (1.00) 30	0.931	72.7 (0.88) 30	0.886
		Yes	78.3 (0.80) 47		72.5 (0.75) 47	
	M	No	77.6 (1.26) 20	0.859	68.8 (1.46) 20	0.039*
		Yes	77.9 (1.37) 17		73.3 (1.49) 17	
	Overall	No	78.0 (0.78) 50	0.911	71.1 (0.83) 50	0.137
		Yes	78.2 (0.69) 64		72.7 (0.67) 64	

Mean (SE) N of examination scores in Years 1 to 4, by gender and self-proposal status.

SP: self-proposal status.

Yes: self-proposed in either Year 2 and/or Year 3.

No: did not self-propose in either Year 2 or Year 3.

OSCE: Objective Structured Clinical Examination.

*significant at p<0.05.

## Discussion

Given the importance afforded to student choice, and the scale of the task of trying to provide genuine choice to all students throughout the entire undergraduate curriculum [5], it would be helpful if it could be shown that student choice results in better academic performance. However, the heterogeneous nature of most SSC programmes means that differences between modules in educational content and in the assessment of students confound interpretation of academic performance in SSC. For these reasons, in the current report, we combined cross-sectional and longitudinal approaches to the analysis of student performance, in SSC, and in standard-set examinations throughout the curriculum.

Our cross-sectional analysis did not show an association between allocated preference and performance in SSC modules. However, firm conclusions cannot be drawn from this about the relationship between student choice and performance in SSC. Our longitudinal analysis suggests that the experience of designing and completing their own SSC modules had only a slight impact overall on student performance in standard-set examinations. Self-proposal status had no impact on performance as a main effect (Table 2); the interaction with gender, although statistically significant, reflected small gender differences in performance between ever and never self-proposers (Table 3). By contrast, the score attained in the first year main standard-set examination was one of the main predictors of subsequent academic performance, and also predicted subsequent self-proposal. Taken together these findings suggest that better-performing students are more likely to self-propose rather than the other way round.

Our study has several strengths. First, our longitudinal analysis allowed us better to assess the causality of observed associations between student choice and academic performance. Second, we assessed performance in standard-set examinations, thus removing a potential confounder. Third, we took into account some of the factors known to affect academic performance, such as gender [9] and graduate status [7]. Fourth, the amount of curriculum time devoted to SSC (one-third) was in line with the recommendations of the original *Tomorrow’s Doctors*[1]; if it had followed the most recent recommendation (10%) [2], a potential criticism may have been that the amount of time was insufficient to allow us to observe an association. Weaknesses include the fact that our study pertains to 2005/06, although the most likely relevant intervening change in undergraduate curriculum planning will have been a reduction in time devoted to SSC (see previous point). Finally, we did not take ethnicity into account in our analyses.

## Conclusion

This is the first study to examine the association between student choice and academic performance. We studied a large, well-characterized cohort of students, and used cross-sectional and longitudinal approaches. Our findings provide little convincing evidence that student choice affects academic performance in our undergraduate curriculum. Rather, they suggest that academically brighter students are more likely to design their own modules.

## Competing interests

The authors declare that they have no competing interests.

## Authors’ contributions

MJM: study concept and design, analysis and interpretation of data, drafting of manuscript. RDeAS: analysis and interpretation of data, critical revision of manuscript for important intellectual content. LC: analysis and interpretation of data, critical revision of manuscript for important intellectual content. MHD: analysis and interpretation of data, critical revision of manuscript for important intellectual content. GJM: critical revision of manuscript for important intellectual content. All authors read and approved the final manuscript.

## Pre-publication history

The pre-publication history for this paper can be accessed here:

http://www.biomedcentral.com/1472-6920/13/26/prepub

## References

[B1] General Medical Council Tomorrow’s Doctors: Recommendations on Undergraduate Medical Education 1993 London: General Medical Council

[B2] General Medical Council Tomorrow’s Doctors: Outcomes and Standards for Undergraduate Medical Education 2009 London: General Medical Council

[B3] Davis MH Harden RM Planning and implementing an undergraduate medical curriculum: the lessons learned Med Teach 2003 25 596 608 10.1080/0142159032000144383 15369907

[B4] Murphy MJ Seneviratne RDA Remers OJ Davis MH ‘Hawks’ and ‘doves’: effect of feedback on grades awarded by supervisors of student selected components Med Teach 2009 31 e484 e488 10.3109/01421590903258670 19877857

[B5] UK Medical Schools - Full list of Quality Assurance Reportshttp://www.gmc-uk.org/education/medical_school_reports_full_list.asp

[B6] Murphy MJ Seneviratne RDA Remers OJ Davis MH Student selected components: student-designed modules are associated with closer alignment of planned and learnt outcomes Med Teach 2009 31 e489 e493 10.3109/01421590903258688 19877858

[B7] Mahesan N Crichton S Sewell H Howell S The effect of an intercalated BSc on subsequent academic performance BMC Med Educ 2011 11 76 10.1186/1472-6920-11-76 21967682 PMC3200165

[B8] Ben-David MF AMEE Guide No.18: Standard setting in student assessment Med Teach 2000 22 120 130 10.1080/01421590078526

[B9] Haist SA Wilson JF Elam CL Blue AV Fosson SE The effect of gender and age on medical school performance: an important interaction Adv Health Sci Educ Theory Pract 2000 5 197 205 10.1023/A:1009829611335 12386462

